# SRSF1 and hnRNP H antagonistically regulate splicing of *COLQ* exon 16 in a congenital myasthenic syndrome

**DOI:** 10.1038/srep13208

**Published:** 2015-08-18

**Authors:** Mohammad Alinoor Rahman, Yoshiteru Azuma, Farhana Nasrin, Jun-ichi Takeda, Mohammad Nazim, Khalid Bin Ahsan, Akio Masuda, Andrew G. Engel, Kinji Ohno

**Affiliations:** 1 Division of Neurogenetics, Center for Neurological Diseases and Cancer, Nagoya University Graduate School of Medicine, Nagoya, Aichi, Japan; 2 Department of Neurology, Mayo Clinic, Rochester, MN, USA

## Abstract

The catalytic subunits of acetylcholinesterase (AChE) are anchored in the basal lamina of the neuromuscular junction using a collagen-like tail subunit (ColQ) encoded by *COLQ*. Mutations in *COLQ* cause endplate AChE deficiency. An A-to-G mutation predicting p.E415G in *COLQ* exon 16 identified in a patient with endplate AChE deficiency causes exclusive skipping of exon 16. RNA affinity purification, mass spectrometry, and siRNA-mediated gene knocking down disclosed that the mutation disrupts binding of a splicing-enhancing RNA-binding protein, SRSF1, and *de novo* gains binding of a splicing-suppressing RNA-binding protein, hnRNP H. MS2-mediated artificial tethering of each factor demonstrated that SRSF1 and hnRNP H antagonistically modulate splicing by binding exclusively to the target in exon 16. Further analyses with artificial mutants revealed that SRSF1 is able to bind to degenerative binding motifs, whereas hnRNP H strictly requires an uninterrupted stretch of poly(G). The mutation compromised splicing of the downstream intron. Isolation of early spliceosome complex revealed that the mutation impairs binding of U1-70K (snRNP70) to the downstream 5′ splice site. Global splicing analysis with RNA-seq revealed that exons carrying the hnRNP H-binding GGGGG motif are predisposed to be skipped compared to those carrying the SRSF1-binding GGAGG motif in both human and mouse brains.

RNA splicing is a highly specialized process especially evolved in humans and other higher metazoans to achieve intricate regulation of gene expressions and to expand the proteome diversity. It is well established that misregulated splicing compromises the fidelity of biological processes and causes a plethora of human diseases. However, the precise molecular mechanisms of how a disease-causing mutation compromises the finely tuned splicing regulation have been dissected in only a limited number of genes. Elucidation of the mechanisms that cause abnormal splicing in human diseases also sheds light on the splicing code in the normal state, and can possibly lead to development of rational therapy.

Congenital myasthenic syndromes (CMSs) are a heterogeneous group of inherited neuromuscular disorders, which arise due to defects in genes encoding presynaptic, synaptic, and postsynaptic proteins expressed at the neuromuscular junction (NMJ)^1^,^2^. Acetylcholinesterase (AChE) encoded by *ACHE* rapidly terminates neuromuscular signal transmission by hydrolyzing the neurotransmitter acetylcholine (ACh). The predominant species of AChE at NMJ is the asymmetric A_12_ species^3^ which comprises three tetramers of the AChE_T_ isoform that are covalently attached to the triple helical collagen-like tail (ColQ). ColQ encoded by *COLQ* is essential for anchoring AChE to the NMJ. ColQ has three distinct domains: an N-terminal proline-rich domain organizing the catalytic AChE subunits into a tetramer, a collagen domain forming a triple helix and harboring two heparan-sulfate-proteoglycan-binding domains (HSPBD), and a C-terminal domain (CTD) enriched in charged amino acids and cysteines. Endplate AChE deficiency is caused by recessive mutations in the *COLQ* gene, but not by mutations in the *ACHE* gene encoding the catalytic subunit^4^. A number of mutations in *COLQ* are associated with endplate AChE deficiency^5^. Based on the position of the mutation and the effect on AChE expression, *COLQ* mutations can fall into four categories^6^: (1) N-terminal mutations compromising the association of AChE_T_ with ColQ; (2) truncation mutations in the collagen domain disrupting the collagenic tail of AChE; (3) CTD missense mutations disrupting triple helical conformation of ColQ; and (4) CTD mutations affecting anchoring of ColQ at NMJ. We exploited specific binding of the HSPBD to perlecan^7^ and of the CTD to MuSK^8^ to develop a protein-anchoring therapy for *Colq*-knockout mice^9^, but there is no rational therapy for human endplate AChE deficiency except for partial mitigation of the symptoms with ephedrine^10^ or albuterol^11^.

Serine/arginine-rich splicing factor 1 (SRSF1) is a ubiquitously expressed splicing factor of the serine (S)- and arginine (R)-rich protein family, which functions in both constitutive and alternative splicing^12^. SRSF1 also has a role in nonsense-mediated mRNA decay (NMD)^13^, mRNA export^14^, and translation^15^. *SRSF1* is also reported to be a proto-oncogene^16^. HnRNP H is a member of heterogeneous nuclear ribonucleoprotein (hnRNP) family, which has been reported to function exclusively in pre-mRNA splicing^17^,^18^,^19^,^20^.

We previously reported a missense mutation (p.E415G) in the CTD of *COLQ* in a patient with endplate AChE deficiency, which causes aberrant skipping of a constitutively spliced exon 16 encoding a part of the ColQ CTD^21^. In this manuscript, we investigate the mechanism underlying aberrant exon skipping. We demonstrate that the mutation disrupts binding of a splicing-enhancing factor SRSF1, and gains a *de novo* binding affinity for a splicing-suppressing factor hnRNP H. We also find that the mutation impairs recruitment of U1 snRNP (U1-70K) to the downstream 5′ splice site.

## Results

### p.E415G in the CTD of ColQ causes skipping of exon 16

**Figure 1:**
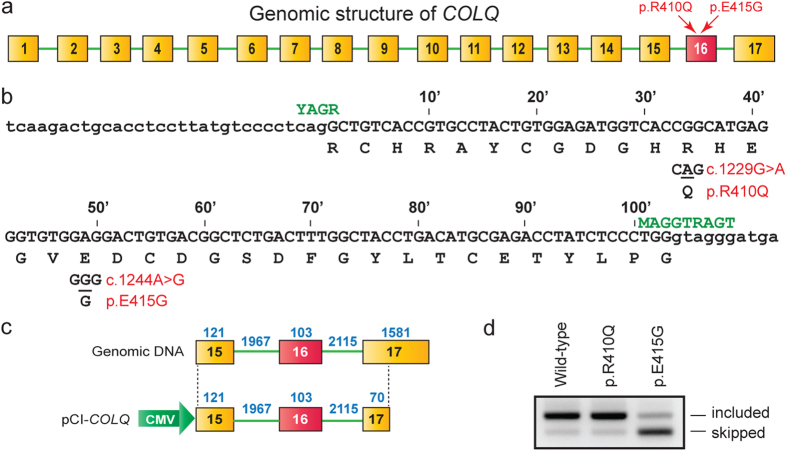
Genomic structure of *COLQ*, identified mutations, and their functional consequences. (**a**) Genomic structure (not drawn to scale) of human *COLQ* gene and two identified mutations in exon 16. (**b**) Nucleotide sequence of *COLQ* exon 16 (uppercase letters) and its flanking introns (lowercase letters). The two mutant nucleotides are underlined. Exonic positions are indicated above the sequence. The consensus sequences of U2-dependent 5′ splice site and 3′ splice site are shown in green letters (Y = C/T, R = A/G, M = A/C)^51^. (**c**) Structure of pCI-*COLQ* minigene. Genomic structure is not drawn to scale. The lengths of exons and introns are indicated in blue. (**d**) RT-PCR of *COLQ* minigene expressed in HeLa cells.

We previously reported two heteroallelic missense mutations in *COLQ* exon 16 in a patient with endplate AChE deficiency (Fig. 1a,b)^21^. We introduced p.R410Q (c.1229G > A) and p.E415G (c.1244A > G) into human *COLQ* cDNA and expressed mutant ColQ proteins in COS cells. We overlaid the purified mutant ColQ on the frog muscle sections, and found that p.R410Q caused loss of binding of ColQ to the frog endplate, whereas p.E415G had no effect on binding of ColQ to the frog endplate^21^. RT-PCR analysis revealed that p.E415G caused skipping of *COLQ* exon 16 in the patient muscle, indicating that p.E415G is not a missense mutation but a splicing mutation. Skipping of exon 16 (103 nt) causes a shift in the reading frame and deletes the C-terminal one-third of the CTD. We reported that similarly deleted or mutated CTDs are incompetent to bind to MuSK and are incapable of being anchored to the NMJ^21^,^22^. In this study, we investigate the molecular basis of aberrant splicing due to p.E415G.

### p.E415G disrupts an exonic splicing enhancer (ESE), and *de novo* generates an exonic splicing silencer (ESS)

**Figure 2:**
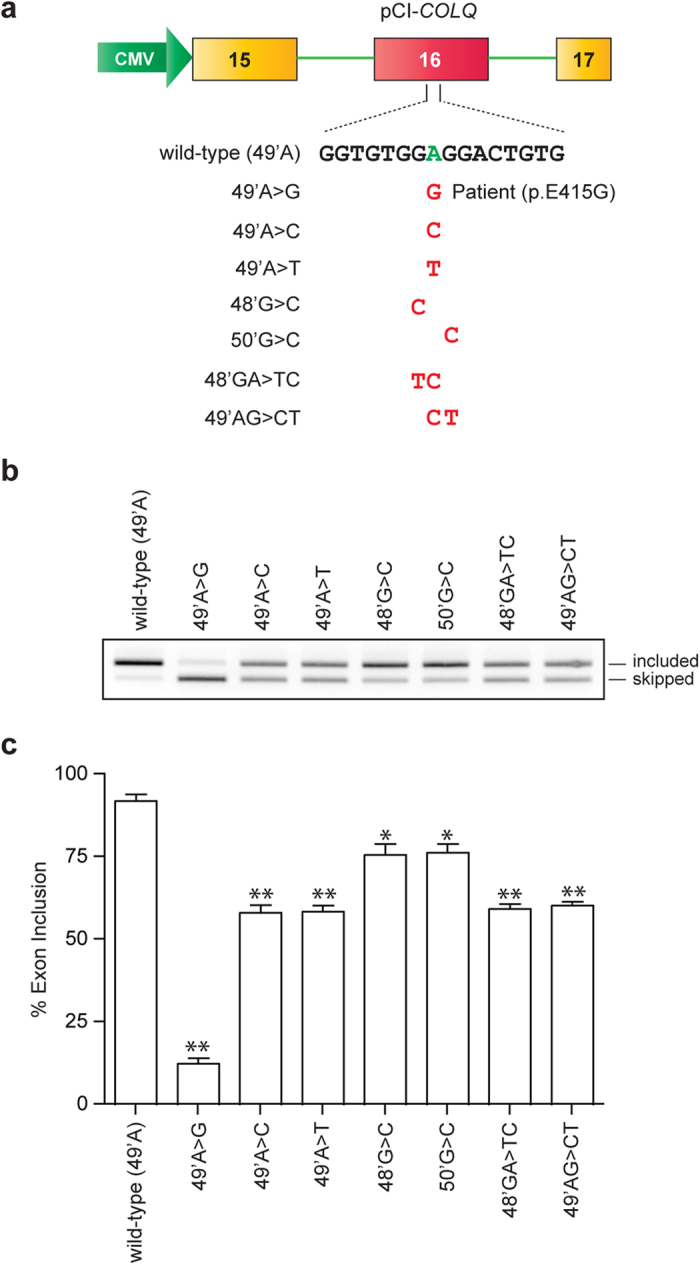
Construction of mutant minigenes and splicing assays. (**a**) Structure of pCI-*COLQ* minigene. The patient’s mutation and six artificial mutations introduced into *COLQ* minigene are indicated. Exonic positions indicated in Fig. 1b are used. (**b**) RT-PCR of *COLQ* minigenes in HeLa cells. (**c**) Ratios of exon 16 inclusion are quantified with image J. Mean and standard deviation (SD) of three independent experiments are indicated. **p < 0.01 and *p < 0.05 compared to the wild-type minigene by Student’s t-test.

We first confirmed that a minigene spanning exons 15 and 17 expressed in HeLa cells recapitulates aberrant splicing (Fig. 1c,d). To examine whether the identified splicing mutation (p.E415G) disrupts an ESE or *de novo* generates an ESS, we engineered six artificial mutations at or around p.E415G (Fig. 2a). All mutants caused skipping of exon 16 with variable degrees (Fig. 2b,c), indicating that the A nucleotide at exonic position 49 and its neighboring nucleotides constitute an ESE. Among the analyzed mutations, the patient’s mutation (G at exonic position 49) caused marked skipping of exon 16 compared to those observed with the other mutants, indicating that the patient’s mutation possibly generates a *de novo* ESS.

### p.E415G disrupts binding of SRSF1 and gains binding of hnRNP H

**Figure 3:**
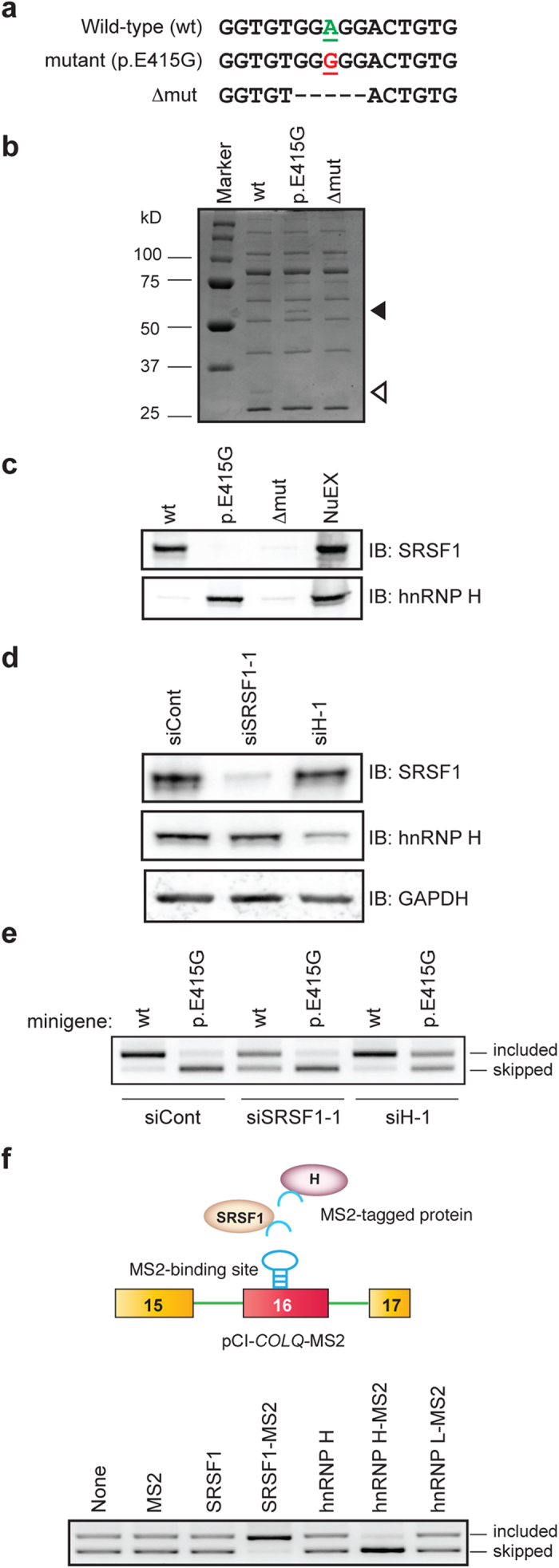
p.E415G compromises binding of a splicing-enhancing SRSF1 and gains binding of a splicing-suppressing hnRNP H. (**a**) Biotinylated RNA probes. (**b**) Coomassie blue staining of RNA affinity-purified products of HeLa nuclear extract. A ~30-kDa protein (open arrowhead) is detected only with the wild-type (wt) probe but not with the p.E415G or deletion mutant (Δmut) probe. In contrast, a ~55-kDa protein (closed arrowhead) is detected only with the p.E415G mutant. (**c**) Immunoblots (IB) of RNA affinity-purified proteins probed with indicated splicing *trans*-factors. The wild-type exon 16 (wt) binds to SRSF1and the p.E415G mutation disrupts its binding. The mutation gains *de novo* binding to hnRNP H. NuEX indicates 5% of the nuclear extract used in the assay. (**d**) Immunoblotting (IB) of HeLa cells treated with siRNA against control (siCont), SRSF1 (siSRSF1-1), and hnRNP H (siH-1) showing efficiency of siSRSF1-1 and siH-1. (**e**) RT-PCR of wild-type (wt) and p.E415G *COLQ* minigenes in HeLa cells treated with indicated siRNAs. A representative gel image of three independent experiments is shown. (**f**) Schematic presentation of a reporter minigene (pCI-*COLQ*-MS2) and *trans*-acting effectors. SRSF1 and hnRNP H (ovals) are fused to the artificial MS2 coat protein (an inverted U shape). MS2 coat protein-binding hairpin RNA is substituted for the splicing regulatory site of exon 16 to directly tether MS2 coat protein-fused SRSF1 and hnRNP H. RT-PCR of pCI-*COLQ*-MS2 minigenes in HeLa cells that are co-transfected with the indicated effectors. A representative gel image of three independent experiments is shown.

Having identified the essential nucleotides that constitute a splicing *cis*-element, we next searched for a splicing *trans*-factor responsible for splicing of exon 16. We employed RNA affinity purification of the HeLa nuclear extract with an RNA probe carrying wild-type or mutant (p.E415G) sequence. Coomassie blue staining of the RNA affinity-purified proteins showed a ~30 kDa protein in the wild-type RNA probe but not in the p.E415G probe (Fig. 3b, open arrowhead). In addition, we noticed *de novo* binding of a ~55 kDa protein in the p.E415G probe (Fig. 3b, closed arrowhead). Mass spectrometry analysis of the excised bands disclosed that the molecule bound to the wild-type RNA probe was SRSF1, and that to the p.E415G RNA probe was hnRNP H. We confirmed the identity of bound proteins by immunoblotting with specific antibodies against SRSF1 (32–4500, Invitrogen) and hnRNP H (A300–511A, Bethyl Laboratories) (Fig. 3c). The immunoblotting indeed demonstrated that SRSF1 binds only to the wild-type probe, whereas hnRNP H binds only to the p.E415G probe.

### SRSF1 enhances and hnRNP H silences inclusion of exon 16

We next examined the effects of SRSF1 and hnRNP H on splicing of exon 16 by siRNA-mediated downregulation of SRSF1 and hnRNP H in HeLa cells. SRSF1 and hnRNP H were efficiently downregulated in HeLa cells (Fig. 3d). Downregulation of SRSF1 induced exclusion of exon 16 in the wild-type minigene (Fig. 3e, lane 3), whereas downregulation of hnRNP H induced inclusion of exon 16 in the p.E415G minigene (Fig. 3e, lane 6), indicating that SRSF1 functions as a splicing enhancer for the wild-type minigene and hnRNP H functions as a splicing silencer for the p.E415G minigene. We observed similar splicing alterations with a second set of siRNAs targeting different sites of each mRNA in HeLa cells (Fig. S1a and b).

We next confirmed that SRSF1 and hnRNP H indeed work on the identified *cis*-element and not on the other sites. To this end, we tethered SRSF1 or hnRNP H to the target using the bacteriophage MS2 coat protein. We made an effector construct expressing MS2-tagged SRSF1 and hnRNP H proteins (MS2-SRSF1 and MS2-H, respectively), and the target minigene construct (pCI-*COLQ*-MS2), in which the MS2-binding site was substituted for the native target site. As expected, tethering of SRSF1 to the target induced inclusion of exon 16 (Fig. 3f, lane 4), whereas tethering of hnRNP H caused skipping of exon 16 (Fig. 3f, lane 6). Lack of the splicing modulating effects of SRSF1 and hnRNP H without the MS2-tag indicates that neither SRSF1 nor hnRNP H works at the other sites (Fig. 3f, lanes 3 and 5). We also confirmed that MS2 alone or MS2-fused hnRNP L (hnRNP L-MS2) had no effect on splicing of pCI-*COLQ*-MS2 (Fig. 3f, lanes 2 and 7). Thus, SRSF1 and hnRNP H exert splicing-enhancing and splicing-silencing activities exclusively on the identified target, respectively.

### Molecular basis of binding of SRSF1 versus hnRNP H and splicing regulation

Previous reports suggest that SRSF1 binds to GA-rich sequence^12^,^23^,^24^ and hnRNP H binds to poly(G) sequence^25^,^26^,^27^. The wild-type *COLQ* exon 16 harbors a motif of GGAGGA, and the patient’s mutation (p.E415G) convert the GGAGG motif to GGGGG. We identified that SRSF1 binds to wild-type RNA probe, whereas hnRNP H binds to the p.E415G RNA probe. The GGAGG motif indeed matches to the functional SELEX consensus of SRSF1 [(G/C)(A/G)(G/C)A(G/C)GA]^28^, as well as to *in vivo* binding motifs 2 and 3 identified by CLIP-seq (cross-linked immunoprecipitation and deep sequencing)^12^. On the other hand, GGGGG motif also matches to the previously identified *in vitro* and *in vivo* binding motif of hnRNP H^25^,^26^,^27^. Therefore, a single nucleotide substitution switches binding of the splicing-enhancing SRSF1 to the splicing-suppressing hnRNP H, and causes aberrant splicing in patient’s muscle.

**Figure 4:**
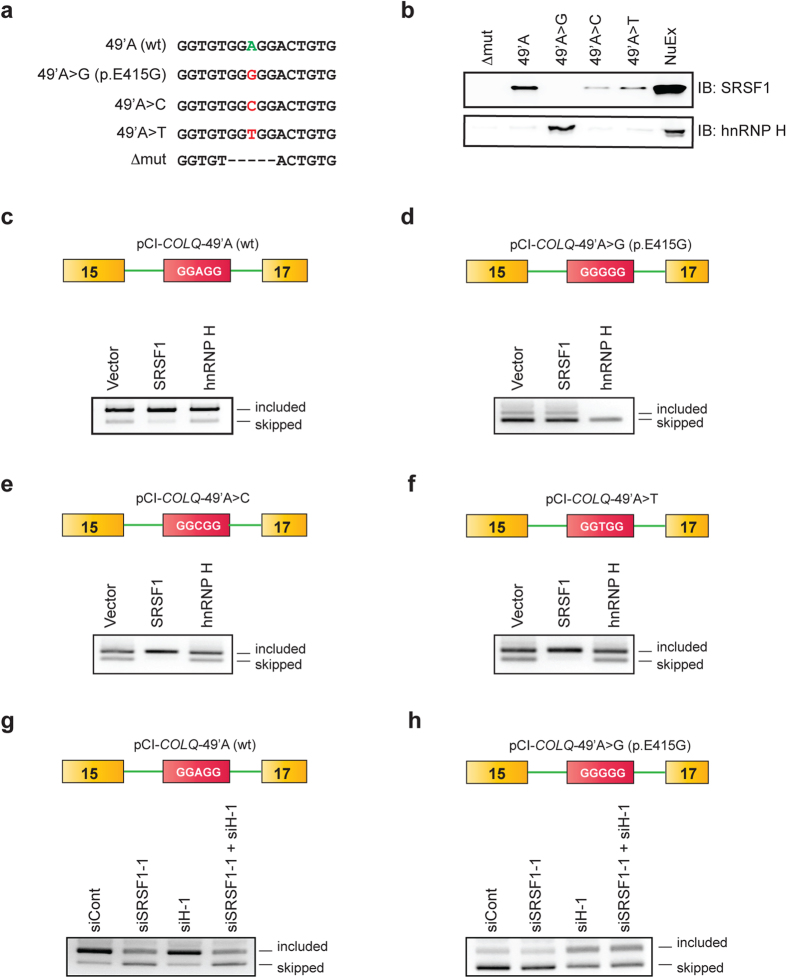
Molecular basis of binding and functional regulation of SRSF1 and hnRNP H. (**a**) Sequences of RNA probes carrying wild-type nucleotide (A), the patient’s mutation (G), and artificial mutations (C, T, and Δmut) in *COLQ* exon 16. (**b**) Immunoblots (IB) of RNA affinity-purified proteins bound to each RNA probe. NuEX indicates 5% of the nuclear extract used in the assay. (**c**–**h**) RT-PCR of pCI-*COLQ* minigenes harboring the indicated motifs that are co-transfected with the indicated *trans*-factors (**c**–**f**) or siRNAs (**g**,**h**) in HeLa cells. Representative gel images of two independent experiments are shown.

We further dissected the molecular basis of splicing regulation by SRSF1 and hnRNP H. Analysis of artificial mutations showed that any mutations affecting the core GGAGG motif affected inclusion of exon 16 (Fig. 2). We therefore examined binding of SRSF1 and hnRNP H to the mutant RNA probes. RNA affinity purification followed by immunoblotting revealed that SRSF1 showed a strong binding affinity for GGAGG, and weak binding affinities for GGTGG and GGCGG (Fig. 4a,b). However, we did not detect binding of SRSF1 to GGGGG. In contrast, hnRNP H showed a strong binding affinity for GGGGG, whereas it could not bind to other motifs. The results of the binding assay were consistent with the splicing analysis of minigenes harboring these motifs (Fig. 2). To further confirm that SRSF1 and hnRNP H indeed regulate splicing of each motif, we overexpressed SRSF1 or hnRNP H in HeLa cells along with a minigene harboring each motif. As expected, SRSF1 exerted an additive effect on exon inclusion for minigenes harboring GGAGG (Fig. 4c), GGCGG (Fig. 4e) and GGTGG (Fig. 4f), to which SRSF1 was able to bind (Fig. 4b). On the contrary, SRSF1 had no effect on a minigene harboring GGGGG (Fig. 4d), to which SRSF1 could not bind. Similarly, overexpression of hnRNP H had no effect on GGAGG (Fig. 4c), GGCGG (Fig. 4e) and GGTGG (Fig. 4f), to which hnRNP H could not bind. In contrast, hnRNP H caused complete skipping of exon 16 harboring GGGGG (Fig. 4d), to which hnRNP H was able to bind (Fig. 4b). Therefore, binding of SRSF1 and hnRNP H to each motif (Fig. 4b) exerted the expected effects on splicing of exon 16 (Fig. 4c–f).

We next asked if there is any binding competition between hnRNP H and SRSF1 for the GGAGG or GGGGG motif, because the G nucleotide in the middle of the motif is an acceptable binding site for SRSF1^12^. For this purpose, we examined splicing of exon 16 of the wild-type (GGAGG) and p.E415G (GGGGG) minigenes after knocking down SRSF1 and/or hnRNP H. For the wild-type minigene, knockdown of SRSF1 alone and knockdown of both SRSF1 and hnRNP H showed similar degrees of exon skipping (Fig. 4g), indicating that hnRNP H had no effect on splicing of exon 16 even in the absence of SRSF1. Similarly, for the p.E415G minigene, SRSF1 had no effect on splicing of exon 16 even in the absence of hnRNP H (Fig. 4h). Taken together, lack of binding of SRSF1 to GGGGG and lack of binding of hnRNP H to GGAGG were not due to competition between SRSF1 and hnRNP H.

### p.E415G impairs splicing of the downstream intron by disrupting SRSF1-induced recruitment of U1 snRNP 70K to the downstream 5′ splice site

**Figure 5:**
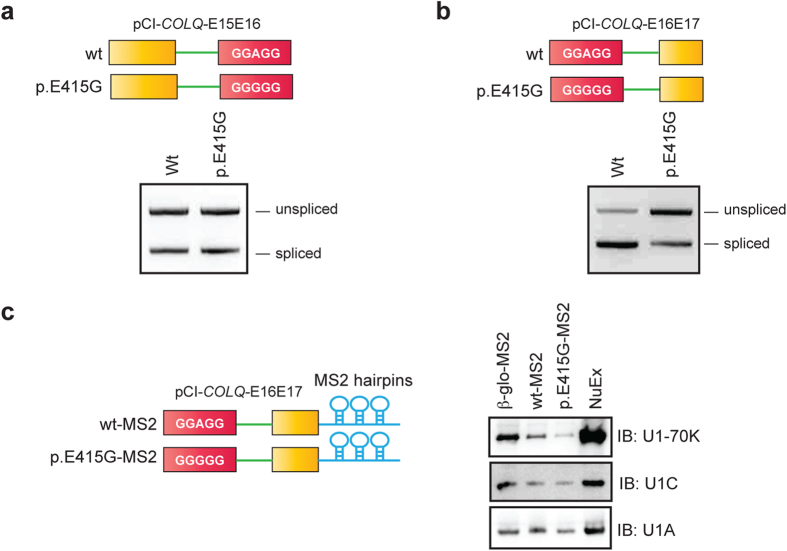
p.E415G compromises recognition of the downstream 5′ splice site by U1-70K. (**a**,**b**) RT-PCR of E15E16 (wt and p.E415G) and E16E17 (wt and p.E415G) minigenes in HeLa cells. (**c**) Schematic structures of MS2-attached wild-type (wt) and p.E415G substrates used for isolation of early spliceosome complex (left). Immunoblotting (IB) of purified spliceosome complex assembled on the indicated substrates (right). β-glo-MS2 is a control construct carrying MS2-attached human β-globin exon 1-intron 1-exon 2^35^. A representative gel image of three independent experiments is shown. Signal intensities of U1-70K bound to wt-MS2 are on average 2.19-fold (SD = 1.21, *n* = 3, *p* < 0.05 by Student’s *t*-test) higher than those bound to p.E415G-MS2.

We next asked if the p.E415G mutation compromises removal of the upstream or downstream intron. We constructed two sets of minigenes, both of which carried either wild-type (wt) or mutant (p.E415G) sequence. The structure of upstream set was “exon 15-intron 15-exon 16” (E15E16-wt and E15E16-p.E415G), and that of downstream set was “exon 16-intron 16-exon 17” (E16E17-wt and E16E17-p.E415G). We examined the splicing efficiency of these minigenes in HeLa cells. We found that both E15E16-wt and E15E16-p.E415G were spliced to a similar extent, suggesting that p.E415G has no effect on splicing of the upstream intron (Fig. 5a). In contrast, splicing of E16E17-p.E415G was inefficient compared to E16E17-wt (Fig. 5b), indicating that p.E415G has an inhibitory effect on splicing of the downstream intron.

We next monitored binding of the associated factors (U1 snRNPs) to the downstream 5′ splice site of the wild-type and p.E415G pre-mRNA substrates. We isolated the early spliceosome complex using the MS2-attached wild-type and p.E415G RNA substrates, and analyzed the associated factors by immunoblotting. We found that E16E17-wt-MS2 and β-globin-MS2 efficiently associated with U1 snRNP 70K (U1-70K) (Fig. 5c, lanes 1 and 2). In contrast, association of U1-70K with E16E17-p.E415G-MS2 was less efficient (Fig. 5c, lane 3). There were no appreciable differences in association of U1 snRNP C (U1C) and U1 snRNP A (U1A) between E16E17-wt-MS2 and E16E17-p.E415G-MS2. These observations along with compromised binding of SRSF1 due to p.E415G suggests that SRSF1 likely promotes recruitment of U1 snRNP 70K to the downstream 5′ splice site to achieve efficient inclusion of exon 16.

### Global effects of the GGGGG and GGAGG motifs in the human and mouse genomes on pre-mRNA splicing

**Figure 6:**
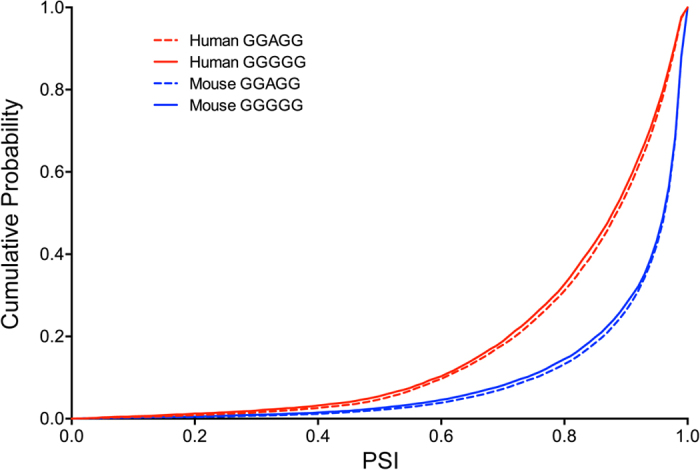
Cumulative distribution function (CDF) plot of PSI’s of exons carrying GGGGG or GGAGG that are expressed in the human and mouse brains. PSI is a ratio of inclusion of each exon in the RNA-seq data. CDF is a fraction of exons with PSI’s of less than or equal to a specific value. PSI’s of exons with GGGGG are shifted to the left compared to those with GGAGG in both human and mouse, indicating that GGGGG-carrying exons are predisposed to be skipped than GGAGG-carrying exons. *P* = 0.00003 between human GGGGG and human GGAGG; *P* = 0.00016 between mouse GGGGG and mouse GGAGG by Student’s *t*-test. Mean and SD are indicated in Table S3.

We next looked into the global significance of the SRSF1-binding GGAGG and hnRNP H-binding GGGGG motifs in the human and mouse genomes. We quantified splicing efficiencies of exons carrying either GGAGG or GGGGG by calculating the percent-spliced-in’s (PSI’s) of RNA-seq data of the human and mouse brains. In both human and mouse, PSI’s of exons carrying GGGGG were significantly lower than those with GGAGG (Fig. 6, Table S3). We also observed a similar tendency in exons flanked by GGGGG- and GGAGG-bearing introns (Fig. S2, Table S3). Thus, a part of human and mouse exons exploit the SRSF1-binding GGAGG and hnRNP H-binding GGGGG motifs to regulate alternative splicing events. It was also interesting to note that human PSI’s were lower than mouse PSI’s, which supports the notion that humans have evolved by acquiring alternative splicing events.

## Discussion

The plasticity and complexity of splicing code enable finely tuned splicing regulation in humans and expand the transcriptome and proteome diversities. However, the increasing splicing complexity predisposes to aberration of splicing regulation that can affect cellular physiology and lead to splicing diseases^29^. Among the numerous splicing codes, ESEs constitute an important class of splicing *cis*-elements that were acquired in the course of evolution. However, ESEs ironically became vulnerable targets of disease-causing mutations. More than 16–20% of the missense mutations of the human mismatch-repair genes hMSH2 and hMLH1 are predicted to disrupt ESEs and affect splicing of the mutant exons^30^. Therefore, molecular dissection of the effects of ESE-disrupting mutations on RNA splicing is essential to understand disease pathomechanisms and to develop rational therapy to correct splicing errors.

We here investigate the underlying mechanisms of a pathogenic splicing mutation in *COLQ* identified in a patient with endplate AChE deficiency, which causes aberrant skipping of a constitutive exon 16^21^. We demonstrate that the mutation disrupts an ESE and *de novo* generates an ESS to induce exclusive exon skipping. We show that the ESE in *COLQ* exon 16 harbors a high affinity-binding motif for SRSF1. Introduction of artificial mutations in the middle of the core GGAGG motif reveals that SRSF1 preferentially binds to ‘A’ but not to ‘G’, ‘C’, or ‘T’. Although binding of SRSF1 to the patient’s ‘G’ mutation is the weakest among the three mutant nucleotides, the difference is marginal. Nevertheless, the ‘G’ mutation markedly causes exon skipping, whereas the effects of artificial ‘C’ and ‘T’ mutations on splicing are moderate. Exclusive binding of hnRNP H to the ‘G’ mutation is likely to have an additional splicing silencing effect and to account for marked skipping of exon 16. We demonstrate that lack of SRSF1 and gain of hnRNP H is the underlying cause of exclusive skipping of exon 16 in the patient’s muscle.

Demonstration of aberrant splicing of *COLQ* not only uncovers the splicing maladies at the patient’s endplates, but also allows us to understand SRSF1-mediated splicing regulation under physiological conditions. To further characterize this regulation in cellular context, we examined splicing of endogenous *COLQ* exon 16 in immortalized human myogenic cells (KD3)^31^,^32^,^33^,^34^ by manipulating the expression levels of SRSF1 and hnRNP H. As expected, overexpression of SRSF1 (Fig. S1c, lane 3) induces inclusion of exon 16 (Fig. S1d, lane 2), whereas overexpression of hnRNP H (Fig. S1c, lane 5) has no effect on splicing of exon 16 (Fig. S1d, lane 3). We also examined the relative expression levels of SRSF1 and hnRNP H in both HeLa and KD3 cells. We found that SRSF1 and hnRNP H are similarly expressed at RNA and protein levels in both KD3 and HeLa cells (Fig. S1e and f). In minigene analysis, we proved that hnRNP H had no effect on splicing of wild-type exon even when SRSF1 is depleted (Fig. 4g). These observations suggest that loss of SRSF1-binding motif (GGAGG) and acquisition of hnRNP H-binding motif (GGGGG) are critical determinants of aberrant skipping *COLQ* exon 16 at the patient’s endplates. The patient may somehow overexpress hnRNP H at the endplates, which may have exacerbated aberrant splicing of *COLQ* exon 16.

Point mutation-mediated transition of splicing *trans*-factors and splicing antagonism between two *trans*-factors is also evident in other genes expressed at the NMJ. We reported a point mutation in *CHRNA1* encoding the acetylcholine receptor (AChR) α subunit in a patient with CMS, which switches binding of a splicing-suppressing RNA-binding protein hnRNP L to a splicing-enhancing RNA-binding protein hnRNP LL^35^. The switch induces inclusion of a non-functional exon P3A into *CHRNA1* transcript, which subsequently nullifies expression of AChR on the cell surface and leads to endplate AChR deficiency. In contrast to hnRNPs L and LL in *CHRNA1*, SRSF1 and hnRNP H do not compete for an identical binding site in *COLQ*. Similar antagonistic splicing regulation is also observed in *SMN1* and *SMN2* pre-mRNAs. *SMN1* and *SMN2* are closely related paralogs with only a single nucleotide substitution (C in *SMN1* and T in *SMN2*) at the 6th nucleotide of exon 7. SRSF1 induces inclusion of exon 7 in *SMN1*^36^,^37^. The C-to-T substitution in *SMN2* gains binding of a splicing-suppressing hnRNP A1^38^,^39^. Additionally, the C-to-T substitution in *SMN2* may^36^,^37^ or may not^38^,^39^ attenuates binding of SRSF1. Thus, antagonistic splicing regulation by binding of antagonistic splicing *trans*-factors to similar motifs can occur in both physiological and pathological conditions.

Dissection of mechanistic basis of splicing of *COLQ* exon 16 reveals that p.E415G disrupts splicing of the downstream intron, but has no effect on splicing of the upstream intron. Analysis of the purified spliceosome complex reveals that p.E415G inhibits the association of U1 snRNPs in early spliceosome complex by disrupting the recruitment of U1-70K in the downstream 5′ splice site. This suggests that SRSF1 probably enhances recognition of the downstream 5′ splice site of *COLQ* exon 16 by U1 snRNP 70K. Previous studies have repeatedly demonstrated that SRSF1 promotes early spliceosome assembly by interacting with U1-70K^40^,^41^,^42^,^43^. The RNA recognition domains (RRM) of SRSF1 promotes the bridging of the RRM of U1-70K to pre-mRNA^40^,^41^,^42^,^43^, thereby allowing multiple binary interactions including RNA-protein, protein-protein, and RNA-RNA, which are essential for the stability of spliceosomal E complex. Therefore, partial dissociation of U1-70K from spliceosome E complex formed on the p.E415G pre-mRNA substrate is likely due to loss of SRSF1-binding. However, we cannot exclude the possibility that hnRNP H exerts an additional inhibitory effect on recruitment of U1 snRNPs to the 5′ splice site.

Having characterized the antagonistic splicing regulation mediated by SRSF1 and hnRNP H that bind to GGAGG and GGGGG, respectively, we next examined the global antagonistic splicing regulation of the GGAGG and GGGGG motifs in the human and mouse genomes. Analysis of RNA-seq data of the human and mouse brains reveals that exons carrying GGGGG have a higher ratio of exon skipping compared to those carrying GGAGG in both human and mouse. Therefore, the hnRNP H-binding GGGGG motif and the SRSF1-binding GGAGG motif are likely to regulate alternative splicing events in some exons. Thus, a single nucleotide substitution that occurred in the course of evolution potentially increases the proteome diversity by activating or suppressing alternative splicing, and the GGGGG and GGAGG motifs are likely to be a representative pair used in alternative regulation of splicing events.

## Materials and Methods

### Patient

The current study was in accord with and approved by the Institutional Review Boards of the Mayo Clinic and Nagoya University Graduate School of Medicine. The patient gave written informed consent to participate in the study. The studies were performed in accordance with the relevant guidelines.

### Cell culture and transfection

HeLa cells were cultured in the Dulbecco’s minimum essential medium (DMEM, Sigma-Aldrich) supplemented with 10% fetal bovine serum (Sigma-Aldrich). HeLa cells were transfected using FuGENE 6 (Roche) according to manufacturer’s instructions, unless otherwise indicated. Immortalized human myogenic cells (KD3) were grown and transfected as described previously^31^.

### Construction of *COLQ* minigene for splicing analysis

To construct a *COLQ* minigene, a 5-kb fragment spanning the 5′ end of exon 15 to the stop codon in exon 17 (Fig. 1c) was amplified by PCR using human genomic DNA isolated from HeLa cells. The forward primer carried an XhoI restriction site followed by the Kozak consensus sequence, 5′-CCACCATG-3′, at the 5′ end. The Kozak sequence was introduced to retain the normal open reading frame of *COLQ*. The reverse primer harbored a NotI restriction site at the 5′ end. The PCR amplicon was cloned into a cytomegalovirus-based expression vector pCI (Promega). The patient’s mutation and artificial mutations were engineered into the pCI minigene using the QuikChange Site-Directed Mutagenesis Kit (Stratagene).

### RT-PCR for splicing analysis

Total RNA was extracted 40 h after transfection using Trizol (Invitrogen), followed by DNase I treatment. cDNA was synthesized with an oligo-dT primer using ReverTra Ace (Toyobo). PCR amplifications were performed by GoTaq (Promega), using primer pairs 5′-CAGCTGACCCCTTTCTACCC-3′ and 5′-AGCGGCAGGGCGTGGAGT-3′.

### RNA affinity purification assay

Biotinylated RNAs were synthesized with the RiboMAX System (Promega) using a PCR-amplified fragment. The PCR-amplicon was generated by annealing two primers followed by overlap extension PCR^44^. Forward primer carried the T7 promoter sequence at the 5′ end. In each 20 μl reaction, 2 μg DNA template was transcribed by T7 RNA polymerase in the presence of 7.5 mM UTP, 7.5 mM ATP, 7.5 mM GTP, 4.5 mM CTP and 3.0 mM Biotin-14-CTP (Invitrogen).

The RNA affinity purification method was slightly modified from the previously reported protocols^35^. Biotinylated RNAs (0.75 nmol) and HeLa nuclear extract (40 μl) (CilBiotech) were mixed in a binding buffer [20 mM HEPES pH 7.8, 150 mM KCl, 0.1 mM EDTA, 1 mM DTT, 1 mM PMSF, 0.05% Triton X, 1×Protease Inhibitor Cocktail (Active Motif)]. A binding reaction of 500 μl was incubated at 30 °C for 2 h with gentle agitation. In parallel, 50 μl streptavidin-conjugated beads (Streptavidin-sepharose, GE Healthcare) were blocked with a 1:1 mixture of 1 ml binding buffer containing yeast tRNA (0.1 mg/100 μl of beads) and 1 ml PBS containing 4% BSA at 4 °C with rotation for 1 h. After blocking, the beads were washed twice in 1 ml binding buffer and mixed with the binding reaction for 2 h at 4 °C with gentle rotation. After washing the beads four times with 1 ml binding buffer, RNA-bound proteins were harvested in SDS loading buffer by boiling at 95 °C for 5 min. The purified proteins were fractionated on a 10% SDS-polyacrylamide gel and stained with Coomassie blue or resolved by immunoblotting.

### Mass spectrometry

The band of interest was excised from the Coomassie blue-stained gel and processed for in-gel digestion by Trypsin Gold (Promega) according to the manufacturer’s protocols. Nanoelectrospray tandem mass analysis was executed using an LCQ Advantage Mass Spectrometry System (Thermo Finnigan) combined with a Paradigm MS4 HPLC System (Michrom BioResources) equipped with a Magic C18AQ column of 0.1 mm in diameter and 50 mm in length (Michrom BioResources). Reversed-phase chromatography was performed with a linear gradient (0 min, 5% B; 45 min, 100% B) of solvent A (2% acetonitrile with 0.1% formic acid) and solvent B (90% acetonitrile with 0.1% formic acid) at an estimated flow rate of 1 μl/min. Ionization was performed with an ADVANCE Captive Spray Source (Michrom BioResources) with a capillary voltage at 1.7 kV and temperature of 150 °C. A precursor ion scan was carried out using a 400–2000 mass to charge ratio (m/z) prior to MS/MS analysis. Multiple MS/MS spectra were resolved by the Mascot program version 2.4.1 (Matrix Science).

### siRNA-mediated knocking down and splicing analysis of minigenes

We synthesized siRNA for human SRSF1 with the sequence of 5′-CCAAGGACAUUGAGGACGUTT-3′ (Sigma Genosys). We previously synthesized siRNA for human hnRNP H^26^. To rule out the possible off-target effects, a second set of siRNAs were similarly synthesized: 5′-GGAAAGAAGAUAUGACCUATT-3′ for human SRSF1 and 5′-GGAAGAAAUUGUUCAGUUC-3′ for human hnRNP H. The control siRNA was AllStar Negative Control siRNA (1027281) by Qiagen.

Cells were plated 24 h before transfection in a six-well culture plate (1.5 × 10^5^ cells/well). The transfection reagent included each siRNA duplex at a final concentration of 30 nM in the Opti-MEM medium, 1 μl Lipofectamine 2000 (Invitrogen), and 500 ng of the minigene in 100 μl DMEM. Three days after incubation at 37 °C, the cells were harvested and were subjected to immunoblotting analysis. Total RNA was also isolated from the harvested cells and RT-PCR was performed for splicing analysis.

### cDNA overexpression and minigene splicing

To construct an expression vector of human SRSF1, we first amplified human SRSF1 cDNA by RT-PCR using total RNA from human skeletal muscle (Clontech). We then cloned the cDNA amplicon into pCDNA3.1D/V5-His TOPO vector (Invitrogen) according to manufacturer’s instructions to obtain pcDNA-SRSF1. The construction of human hnRNP H cDNA in pCDNA3.1D/V5-His TOPO vector (pcDNA-hnRNP H) was previously described^26^. Prior to transfection, cells were plated 24 h in a six-well culture plate (1.5 × 10^5^ cells/well) and transfected with 1 μg of the expression construct, 500 ng of the minigene, and 3.5 μl of FuGENE 6 (Roche) in 100 μl Opti-MEM medium according to the manufacturer’s instructions. Three days after incubation at 37 °C, the cells were harvested and were subjected to immunoblotting analysis. For splicing analysis, total RNA was also isolated from the harvested cells and RT-PCR was performed.

### Harvesting cells for immunoblotting

Cells were washed twice in PBS and harvested in PBS with 1×Protease Inhibitor Cocktail. After centrifugation at 2,000 × *g* for 5 min, the pellets were suspended in buffer A (10 mM HEPES-NaOH pH 7.8, 10 mM KCl, 0.1 mM EDTA, 1 mM DTT, 0.5 mM PMSF, 0.1% Nonidet P-40, 1×Protease Inhibitor Cocktail) and kept for 30 min on ice. Following sonication, samples were centrifuged at 20,000 × *g* for 5 min. The supernatants were collected as total cell lysate.

### MS2-mediated artificial tethering of *trans*-factor

Artificial tethering was performed by co-transfection of a reporter minigene and an effector construct as previously described^35^. We introduced the MS2-binding site (5′-ACATGAGGATCACCCATGT-3′)^35^ in the minigene by replacing the native target using the QuikChange Site-Directed Mutagenesis Kit, so that effector molecule can bind to the artificially inserted target site in the reporter minigene. To construct pcDNA-SRSF1-MS2, an insert encoding MS2 was isolated from pcDNA-hnRNP L-MS2^35^ using XhoI and XbaI restriction enzymes, purified, and cloned into the respective sites of pcDNA-SRSF1. We previously made pcDNA-hnRNP H-MS2 expressing hnRNP H-MS2^26^ and pcDNA-MS2 expressing MS2 alone^35^.

### Splice site function and early spliceosomal complex assays

We constructed E15E16 (wt and p.E415G) minigenes spanning *COLQ* exon 15 to 16 and E16E17 (wt and p.E415G) minigenes spanning *COLQ* exon 16 to 17 in pcDNA3.1D/V5-His-TOPO vector (Invitrogen). Amplicons were generated by PCR using pCI-*COLQ* (wt or p.E415G), and cloned into pcDNA3.1D/V5-His-TOPO vector.

We introduced three copies MS2-coat protein binding hairpin sequences at the 3′ end of E16E17 (wt and E415G) constructs using the megaprimer method^45^. At first, we PCR-amplified a fragment harboring three copies MS2-coat protein binding hairpin sequences from pSP64-MS2 vector^35^ with the primers carrying complementary sequences to E16E17 minigene where the MS2-sequences is being inserted. The PCR amplicon was used as a megaprimer for the QuikChange site-directed mutagenesis system. These vectors were used as templates to generate MS2-attached RNA substrates of E16E17 (wt and p.E415G) for isolation of early spliceosomal complex. As control, we used MS2-attached human β-globin exon 1-intron 1-exon 2 construct (pSP64-HβΔ6-MS2) as previously described^35^.

### MS2-affinity isolation of early spliceosomal complex

One pmol of the RNA probe (β-globin-MS2, E16E17-wt-MS2, or E16E17-p.E415G-MS2) was incubated with a 20-fold molar excess of MS2-MBP fusion protein^46^ before mixing it with HeLa nuclear extract. Fifty μl of HeLa nuclear extract was pretreated with 10 μl (bead volume) of amylose resin (New England Biolabs) overnight at 4 °C. The pretreated HeLa nuclear extract was incubated at 37 °C for 30 min with a mixture of the RNA probe and the MS2-MBP fusion protein at final concentrations of 60 mM KCl and 25% HeLa nuclear extract. Then amylose resin beads (20 μl) was added in the mixture and incubated at 4 °C for 30 min with gentle rotation. After washing the resin four times with wash a buffer (20 mM HEPES pH 8.0, 150 mM KCl, and 0.05% Triton X-100), bound proteins were eluted with 10 mM maltose solution and subjected to SDS-PAGE followed by immunoblot analyses.

### Analysis of the GGGGG and GGAGG motifs in the human and mouse genomes

To understand the effects of the SRSF1-binding GGAGG motif and hnRNP H-binding GGGGG motif on pre-mRNA splicing in the human and mouse genomes, we analyzed RNA-seq of the brains of human (Illumina BodyMap 2.0 at http://www.ebi.ac.uk/arrayexpress/experiments/E-MTAB-513/) and mouse^47^. RNA-seq of human and mouse brains had 63,966,169 and 93,246,802 paired-end reads, respectively. RNA-seq fastq files were mapped to the human genome hg19/GRCh37 or the mouse genome mm9 using TopHat version 2.0.12^48^. The mapping efficiency was 87.0% and 78.5%, respectively. The mapped reads were analyzed at the transcript level with Cufflinks version 2.2.1^49^. Among 217,852 and 206,107 exons annotated in Ensembl release 65, 114,971 (52.8%) and 128,785 (62.5%) exons were expressed in the human and mouse brains, respectively. The numbers of GGGGG- and GGAGG-bearing exons among the expressed exons are shown in Supplementary Table S1. The copy numbers of GGGGG- and GGAGG-motifs within an exon are shown in Supplementary Table S2. The percent-spliced-in (PSI) values of the expressed exons carrying GGGGG or GGAGG were calculated using MISO version 0.5.2^50^. We compared PSI values of the motif-bearing and motif-lacking exons. Cumulative distribution functions were plotted with Prism 6.0f (GraphPad software).

### Antibodies

Antibodies used in this study were anti-SRSF1 (32–4500, Invitrogen), anti-U1-snRNP 70K (U1-70K) (H111, kindly provided by Akila Mayeda, Division of Gene Expression Mechanism, Fujita Health University), anti-U1 snRNP C (U1C) (4H12, Sigma-Aldrich), anti-U1 snRNP A (U1A) (PA5-27474, Thermo Fisher Scientific Pierce), anti-His-tag (D293-1, Medical & Biological Laboratories) and anti-GAPDH (Sigma-Aldrich).

## Additional Information

**How to cite this article**: Rahman, M. A. *et al.* SRSF1 and hnRNP H antagonistically regulate splicing of *COLQ* exon 16 in a congenital myasthenic syndrome. *Sci. Rep.* **5**, 13208; doi: 10.1038/srep13208 (2015).

## Supplementary Material

Supplementary Information
