# Supplementary material for: SRSF1 and hnRNP H antagonistically regulate splicing of COLQ exon 16 in a congenital myasthenic syndrome
Source: Sci Rep. 2015 Aug 18;5:13208. doi: 10.1038/srep13208 (PMC4539547; doi:10.1038/srep13208)
Supplement: Supplementary Information [file srep13208-s1.pdf]

Supplementary Information

SRSF1 and hnRNP H antagonistically regulate splicing of *COLQ* exon 16 in a congenital myasthenic syndrome

Mohammad Alinoor Rahman[1], Yoshiteru Azuma[1], Farhana Nasrin[1], Jun-ichi Takeda[1], Mohammad Nazim[1], Khalid Bin Ahsan[1], Akio Masuda[1], Andrew G. Engel[2], and Kinji Ohno[1]

[1]Division of Neurogenetics, Center for Neurological Diseases and Cancer, Nagoya University Graduate School of Medicine, Nagoya, Aichi, Japan
[2]Department of Neurology, Mayo Clinic, Rochester, MN, USA

Address correspondence to: Kinji Ohno, Division of Neurogenetics, Center for Neurological Diseases and Cancer, Nagoya University Graduate School of Medicine, 65 Tsurumai, Showa-ku, Nagoya 466-8550, Japan
Phone: +81-52-744-2446, Fax: +81-52-744-2449, e-mail: ohnok@med.nagoya-u.ac.jp

Supplementary information includes:

Supplementary Figures S1-S2

Supplementary Tables S1-S3

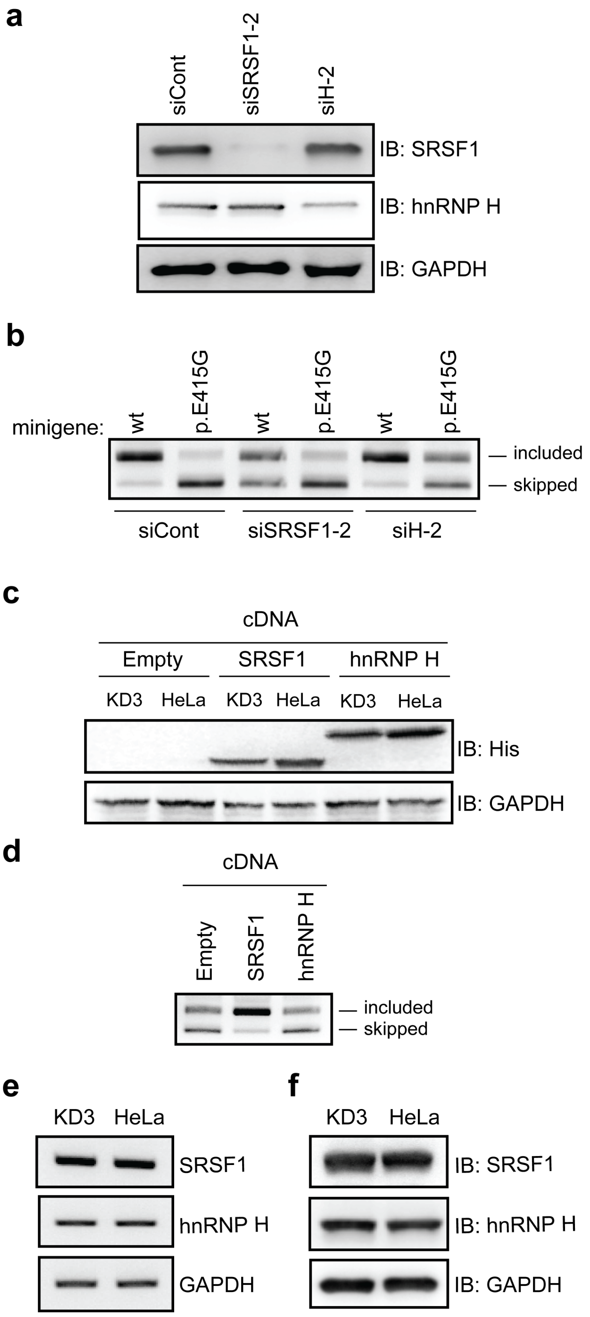

Supplementary Figure S1. Additional data related to Figures 3 and 4. (a) Immunoblotting (IB) of HeLa cells treated with siRNA against control (siCont), SRSF1 (siSRSF1-2), and hnRNP H (siH-2). (b) RT-PCR of wild-type (wt) and p.E415G *COLQ* minigenes in HeLa cells treated with indicated siRNAs. (c) Immunoblotting (IB) to detect over expressions of indicated cDNA constructs in KD3 and HeLa cells. (d) Splicing RT-PCR of endogenous *COLQ* exon 16 in KD3 cells that overexpress the indicated *trans*-factors. (e and f) Endogenous expression of SRSF1 and hnRNP H in KD3 and HeLa cells detected by RT-PCR (e) and Western blotting (f). Each panel is a representative image of three independent experiments.

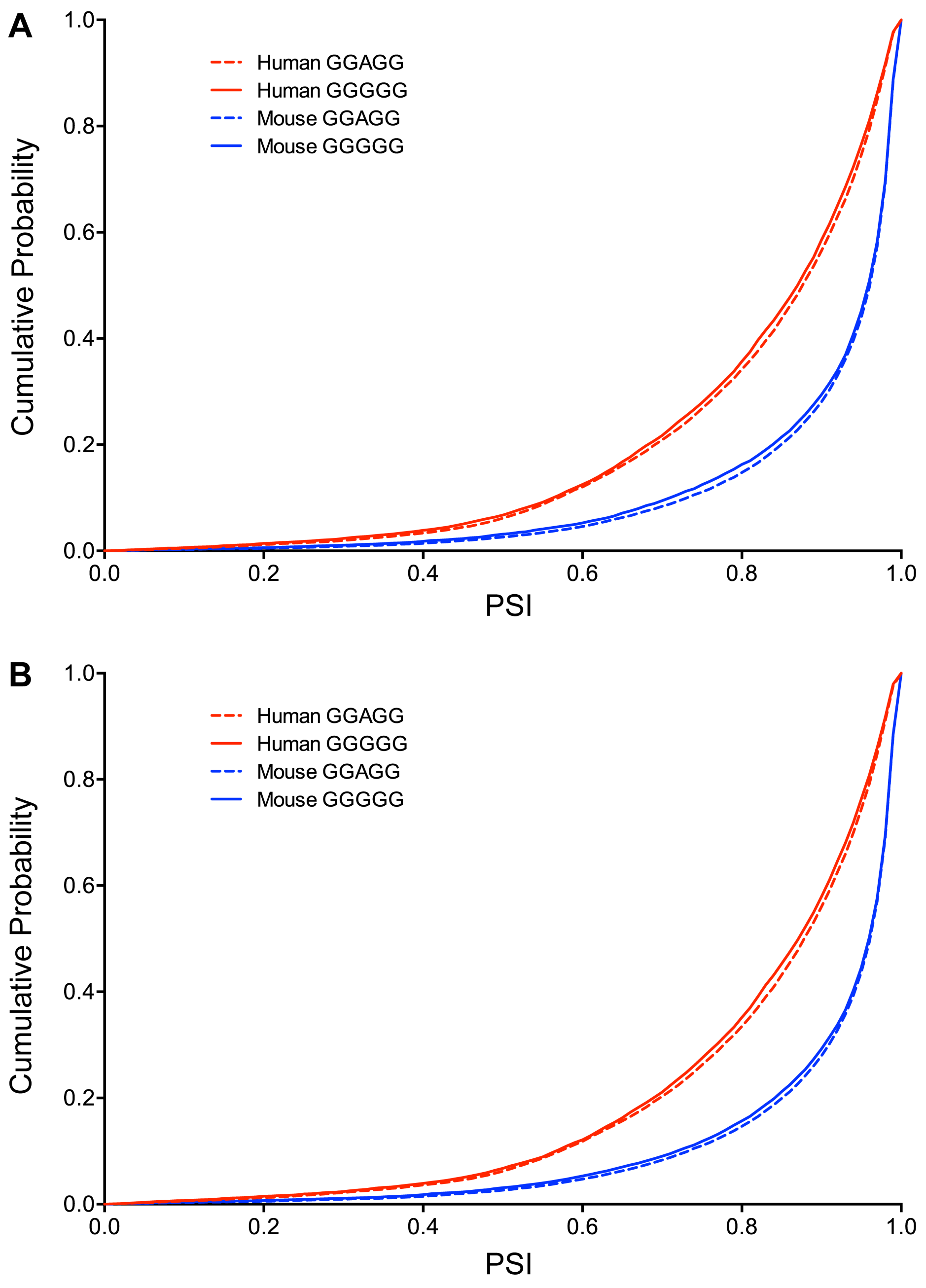

Supplementary Figure S2. Cumulative distribution function (CDF) plots of PSI's of exons with GGGGG- or GGAGG-motif(s) either in the upstream (A) or downstream (B) intron. The analysis is similar to Figure 6, in which exonic motifs are analyzed. In both human and mouse brains, PSI's of exons with GGGGG in the upstream and downstream intron are shifted to the left compared to those with GGAGG, indicating that GGGGG in the flanking intron leads to skipping of the exon compared to GGAGG in the flanking intron. $P = 0.00408$ and $P = 0.00083$ between human GGGGG and human GGAGG in the upstream and downstream introns, respectively; $P = 0.00013$ and $P = 0.00205$ between mouse GGGGG and mouse GGAGG in the upstream and downstream introns, respectively, by Student's *t*-test. Mean and SD are indicated in Supplementary Table S3.

Supplementary Table S1. The number of GGGGG- and GGAGG-bearing exons expressed in the human and mouse brain

Motif	GGGGG	GGAGG
Human	11,342[a]/114,971[b] (10.2%)	24,497[a]/114,971[b] (22.1%)
Mouse	11,301[a]/128,785[b] (8.9%)	27,481[a]/128,785[b] (21.8%)

[a]The number of motif-bearing exons among exons expressed in the brain. [b]The number of exons expressed in the brain. The annotation is based on Ensemble release 65 for both human and mouse. RNA-seq of the brains of human (Illumina BodyMap 2.0 at http://www.ebi.ac.uk/arrayexpress/experiments/E-MTAB-513/) and mouse[42] are analyzed.

Supplementary Table S2. The number of copies of GGGGG- and GGAGG-motifs in a single exon

	Human		Mouse	
Copies	GGGGG	GGAGG	GGGGG	GGAGG
1	7,631	17,736	8,023	20,444
2	2,183	4,234	2,150	4,515
3	765	1,163	610	1,233
4	336	531	209	523
≥ 5	427	833	309	766
Mean ± SD	1.67 ± 2.43	1.57 ± 2.34	1.53 ± 1.81	1.50 ± 1.91

The number of exons harboring the indicated copy number of motifs is shown. Only exons expressed in the brain (see Supplementary Table S1) are analyzed. Mean and SD of the copy numbers are indicated at the bottom.

Supplementary Table S3. Mean and SD of PSI's of exons carrying either GGAGG or GGGGG, as well as of exons flanked by introns carrying GGAGG or GGGGG

	Human			Mouse		
	GGAGG	GGGGG	*p*	GGAGG	GGGGG	*p*
Exon	0.836 ± 0.165	0.828 ± 0.172	0.00003	0.915 ± 0.130	0.909 ± 0.138	0.00016
Upstream intron	0.822 ± 0.177	0.815 ± 0.181	0.00408	0.908 ± 0.138	0.902 ± 0.146	0.00013
Downstream intron	0.824 ± 0.178	0.817 ± 0.182	0.00083	0.908 ± 0.139	0.903 ± 0.146	0.00205

Cumulative distribution function plots of individual data are shown in Fig. 6 and Supplementary Fig. S1. Statistical difference is calculated by Student's *t*-test.
